# A dual role for Caspase8 and NF-*κ*B interactions in regulating apoptosis and necroptosis of ovarian cancer, with correlation to patient survival

**DOI:** 10.1038/cddiscovery.2015.53

**Published:** 2015-12-14

**Authors:** L Hernandez, M K Kim, A M Noonan, E Sagher, H Kohlhammer, G Wright, L T Lyle, P S Steeg, M Anver, D D Bowtell, on behalf of the Australian Ovarian Cancer Study Group ,, C M Annunziata

**Affiliations:** 1 Women’s Malignancies Branch, National Cancer Institute, Bethesda, MD 20892-1906, USA; 2 Metabolism Branch, Center for Cancer Research, National Cancer Institute, Bethesda, MD 20892-1906, USA; 3 Biometric Research Branch, Division of Cancer Treatment and Diagnosis, National Cancer Institute, Bethesda, MD 20892-1906, USA; 4 Pathology/Histotechnology Laboratory, LASP, Leidos Biomedical Research, Inc., Frederick, MD 21702-1201, USA; 5 Centre for Cancer Genomics and Predictive Medicine, Peter MacCallum Cancer Centre, East Melbourne, Victoria, Australia; 6 The Department of Pathology, University of Melbourne, Parkville, Victoria, Australia

## Abstract

Ovarian cancer is a deadly disease characterized by primary and acquired resistance to chemotherapy. We previously associated NF-*κ*B signaling with poor survival in ovarian cancer, and functionally demonstrated this pathway as mediating proliferation, invasion and metastasis. We aimed to identify cooperating pathways in NF-*κ*B-dependent ovarian cancer cells, using genome-wide RNA interference as a loss-of-function screen for key regulators of cell survival with IKK*β* inhibition. Functional genomic screen for interactions with NF-*κ*B in ovarian cancer showed that cells depleted of Caspase8 died better with IKK*β* inhibition. Overall, low Caspase8 was associated with shorter overall survival in three independent gene expression data sets of ovarian cancers. Conversely, Caspase8 expression was markedly highest in ovarian cancer subtypes characterized by strong T-cell infiltration and better overall prognosis, suggesting that Caspase8 expression increased chemotherapy-induced cell death. We investigated the effects of Caspase8 depletion on apoptosis and necroptosis of TNF*α*-stimulated ovarian cancer cell lines. Inhibition of NF-*κ*B in ovarian cancer cells switched the effects of TNF*α* signaling from proliferation to death. Although Caspase8-high cancer cells died by apoptosis, Caspase8 depletion downregulated NF-*κ*B signaling, stabilized RIPK1 and promoted necroptotic cell death. Blockage of NF-*κ*B signaling and depletion of cIAP with SMAC-mimetic further rendered these cells susceptible to killing by necroptosis. These findings have implications for anticancer strategies to improve outcome for women with low Caspase8-expressing ovarian cancer.

## Introduction

Ovarian cancer is the most lethal gynecological cancer in the United States.^1^ Molecular profiling identified subtypes with prognostic implications, suggesting novel therapeutic targets.^2–5^ The Cancer Genome Atlas (TCGA) defined subtypes of ovarian cancer: differentiated, immunoreactive, mesenchymal, proliferative.^3^ These mirrored the subtypes defined by the Australian Ovarian Cancer Study (AOCS).^4^ These two independent studies identified immune-related subtypes, with strong T-cell infiltrate and expression of genes involved in inflammation.

NF-*κ*B regulates inflammation,^6,7^ and we recently demonstrated the biological relevance of NF-*κ*B in ovarian cancer. Coordinated expression of NF-*κ*B transcription factors and target genes were associated with poor overall survival and aggressive tumor behavior.^8,9^ As constitutive NF-*κ*B signaling defines a subset of ovarian cancer dependent on this pathway, we hypothesized that targeting compensatory pathways may provide additional therapeutic benefit.

TNF*α*-induced NF-*κ*B signaling is a well-known survival pathway in cancer cells.^10,11^ TNF*α* exerts pro-survival effects in ovarian cancer.^12,13^ TNF*α* pathways are best studied in inflammatory cells, where receptor binding prompts complex formation between RIPK1 and cIAP proteins, activating NF-*κ*B. Subsequent upregulation of proinflammatory cytokines such as IL-8, and inhibitors of apoptosis cIAP1 and cFLIP, interact with Caspase8 and limit its pro-apoptotic activity.^14^

The induction of cell death is a goal of cancer therapy, and NF-*κ*B is a mechanism to avert death and promote growth. IAPs suppress programmed cell death, but are neutralized by SMAC during apoptosis. Thus SMAC-mimetics are useful antagonists to reverse the suppression of caspases.^15^ When NF-*κ*B is blocked, TNF receptor engagement activates two forms of cell death.^16^ In one instance, when cFLIP-p43 expression and NF-*κ*B signaling are low, Caspase8 homodimerizes and cleaves RIPK1 to trigger apoptosis. When Caspase8 is functionally absent and NF-*κ*B signaling is inactive, uncleaved RIPK1 induces necroptosis.^17,18^

The current study utilized a genome-wide RNA interference approach to identify candidate genes responsible for ovarian cancer cell survival in the context of NF-*κ*B signaling. The loss-of-function screen in combination with IKK*β* inhibition identified an interplay between Caspase8 and NF-*κ*B in ovarian cancer. Here we describe a dual role for Caspase8 in ovarian cancer, and demonstrate a relationship with outcome in genomic analyses.

## Results

### Caspase8 depletion sensitizes Ovcar3 cells to IKK*β* inhibition

We previously showed that blocking IKK*β* significantly decreased viability of ovarian cancer cell lines.^9^ Ovcar3 cells represent NF-*κ*B-dependent ovarian cancers, as IKK*β* inhibitor decreased their viability. Reporter assay confirmed dose-dependent inhibition of constitutive NF-*κ*B transcriptional activity in Ovcar3. Near complete inhibition was achieved at 2.5 *μ*M of a specific IKK*β* inhibitor (Supplementary Figure S1A). This inhibitor moderately (17%) decreased Ovcar3 viability after 3 days, whereas there was 70% loss after 7 days (Supplementary Figure S1B) suggesting that sustained NF-*κ*B inhibition was required for growth inhibition.

**Figure 1:**
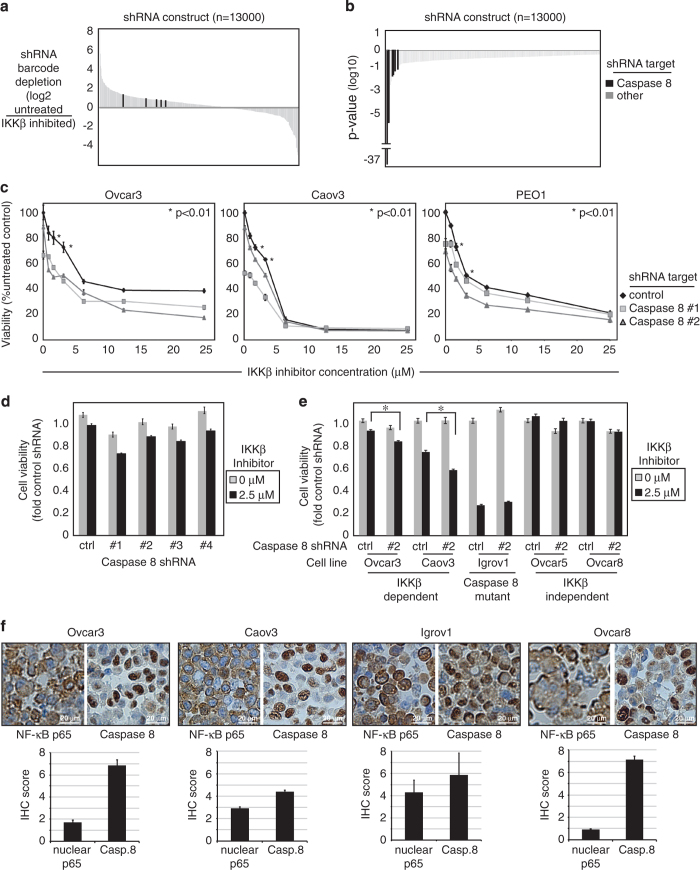
Caspase8 inhibition compounds cytotoxicity in ovarian cancer cells treated with IKK*β* inhibitor. Caspase8 shRNA toxicity in a sensitization library screen is shown as (**a**) the log2 ratio of untreated *versus* IKK*β*-inhibited, or (**b**) log 10 *P*-value, after 10 days. Caspase8 shRNAs are highlighted in black. (**c**) Cell viability of Ovcar3, Caov3 and PEO1 cells transduced with control shRNA or either of two different Caspase8 shRNAs was measured by XTT after 7 days of exposure to increasing concentrations of IKK*β* inhibitor. Data are shown as fold control shRNA, in the absence of IKK*β* inhibitor (DMSO), ±S.E.M., *n*=8. Asterisks indicate significant differences (*P*<0.01) at the concentration range used in subsequent studies. (**d**) Ovcar3 cells were transduced with Caspase8 shRNAs #1, #2, #3 or #4 or control shRNA and exposed to 2.5 *μ*M IKK*β* inhibitor or vehicle for 7 days. Viability was measured by XTT and is shown as fold control shRNA and drug control (DMSO). Error bars represent S.E.M., *n*=8; *P<0.001. (**e**) Ovcar3 cells expressing control or Caspase8 shRNA #2 were treated with 2.5 *μ*M IKK*β* inhibitor for 7 days. (**f**) Three ovarian cell lines sensitive to IKK*β* inhibition (Ovcar3, Caov3 and Igrov1) and one insensitive cell line (Ovcar8) were stained by IHC with NF-*κ*B-p65 and Caspase8 antibodies and the presence of nuclear protein analyzed. IHC scores for relative amounts of nuclear NF-*κ*B-p65 and Caspase8 were measured in four to six uniform fields per sample and averaged. Numbers are average scores ±S.E.M.

To identify pathways that cooperate with IKK*β*, we performed a genomic shRNA screen at time points greater than 7 days. Ovcar3 cells were grown for 10 or 14 days following shRNA library expression in the presence of sublethal concentration of IKK*β* inhibitor or vehicle. Knockdown of specific genes significantly sensitized cells to IKK*β* inhibitor in four replicate experiments (>0.6-fold decreased, *P*<0.05; Supplementary Table 1). In two independent quadruplicate experiments, Caspase8 shRNAs most reproducibly decreased survival with IKK*β* inhibitor (Figure 1a). Each of the five different shRNA constructs against Caspase8 significantly decreased Ovcar3 viability with IKK*β* inhibitor compared with control (Figure 1b, *P*<0.04 to *P*<10^−37^). Caspase8 shRNAs significantly decreased cell viability in combination with IKK*β* inhibitor in three ovarian cancer cell lines, especially at low concentrations (Figure 1c, *P*<0.01). Four shRNAs common to both independent experiments individually reproduced the additive effects with IKK*β* inhibitor (Figure 1d, and Supplementary Table 2). All the four shRNAs depleted Caspase8 mRNA expression by 40–60%, maintained for 10 days, producing comparable reduction in protein (Supplementary Figures S2A and B). Caspase8 depletion or IKK*β* inhibitor at low concentration had minimal effects on cell viability, but in the context of IKK*β* inhibitor, each Caspase8 shRNA further reduced cell viability compared with control (Figure 1d).

We tested combined Caspase8 depletion and IKK*β* inhibitor in additional cell lines shown to be sensitive or resistant to IKK*β* inhibition.^9^ Ovcar3 and Caov3 cells are sensitive to IKK*β* inhibitor, and showed additionally decreased viability with Caspase8 depleted, an effect evident even at low IKK*β-*inhibitor concentrations (*P*<0.001, Figure 1e). Consistent with this effect, Caspase8-mutant cell line Igrov1 was extremely sensitive to IKK*β* inhibitor, and this was not enhanced by Caspase8 shRNA (Figure 1e). Conversely, in Ovcar5 and Ovcar8 cells, shown to be relatively resistant to IKK*β* inhibitor,^9^ IKK*β*-inhibitor and Caspase8 depletion had little effect (Figure 1e). These results emphasize the cell-type specificity of cooperation between Caspase8 and NF-*κ*B signaling in ovarian cancer cells.

Caspase8 has an enzymatic function, cleaving itself and other proteins during apoptosis. To investigate whether the combined lethality depended on Caspase8 enzymatic activity, we assessed Ovcar3 viability with Caspase8 inhibitor ZIETD, in the absence and presence of IKK*β* inhibitor (Supplementary Figure S3). Caspase enzyme inhibition over 7 days did not affect the viability. IKK*β* inhibitor reduced the viability in a dose-dependent manner. Dual inhibition of Caspase8 and IKK*β* did not increase cell death over IKK*β* inhibitor alone, suggesting that Caspase8 enzymatic activity was not responsible for its cooperation with IKK*β*.

Protein expression of Caspase8 and NF-*κ*B transcription factor p65 was examined by immunohistochemistry in ovarian cancer cell lines. Nuclear p65 suggests NF-*κ*B activation, and this was relatively higher in sensitive cell lines, compared with insensitive cell line Ovcar8 (Figure 1f). Caspase8 was present in all the cell lines, but demonstrated an unusual peri-nuclear pattern in Igrov1, where it is mutated.^19^ Thus, for studies of Caspase8 biologic effects in ovarian cancer, we established an isogenic pair of Ovcar3, with either endogenous high Caspase8 or stable knockdown of Caspase8.

### Caspase8 depletion decreased NF-*κ*B transcriptional activity

**Figure 2:**
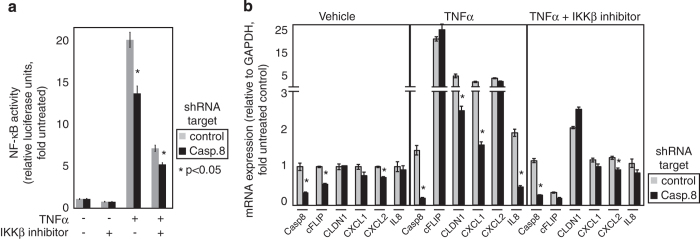
IKK*β* inhibition downregulates NF-*κ*B signaling and caspase activation after TNF*α* stimulation. (**a**) Ovcar3 cells expressing control or Caspase8 shRNA were transduced with NF-*κ*B luciferase reporter. NF-*κ*B signaling in was measured after 18 h treatment with TNF*α* (10 ng/ml) and/or IKK*β* inhibitor (2.5 *μ*M). Data are arbitrary luciferase units normalized to XTT and are represented as mean±S.E.M.; *P<0.05 based on *t*-test. (**b**) mRNA expression of Caspase8 and NF-*κ*B target genes cFLIP, CLDN1, CXCL1, CXCL2 and IL-8 was measured by quantitative RT-PCR in Ovcar3 cells, expressing either control or Caspase8 shRNA. Expression was normalized to that of GAPDH, and expressed as mean±S.E.M. of triplicate experiments. *P<0.05 based on *t*-test.

Ovarian cancer patients express elevated serum TNF*α*,^20^ which can promote tumor cell death, or enhance NF-*κ*B signaling, depending on cellular context. Given our shRNA screen results, we hypothesized that Caspase8 might support NF-*κ*B signaling in ovarian cancer. TNF*α* stimulation of Ovcar3 stably expressing NF-*κ*B luciferase reporter produced nearly 20-fold activation of NF-*κ*B transcriptional activity, which was diminished by IKK*β* inhibitor (Figure 2a). Caspase8 depletion attenuated TNF*α*-induced NF-*κ*B activity by 20–30% (*P*<0.05), highlighting the positive role of Caspase8 in NF-*κ*B signaling. Caspase8 depletion decreased endogenous mRNA expression of our previously established ovarian cancer NF-*κ*B signature,^9,21^ and/or attenuated the increase after TNF*α* stimulation (Figure 2b, *P*<0.05). As a control, IKK*β* inhibitor blocked the rise of these genes, and Caspase8 knockdown had little additional effect. This suggested that Caspase8 depletion negatively affected NF-*κ*B transcriptional activity in ovarian cancer cells.

### Caspase8 expression and NF-*κ*B in subtypes of human ovarian cancers

**Figure 3:**
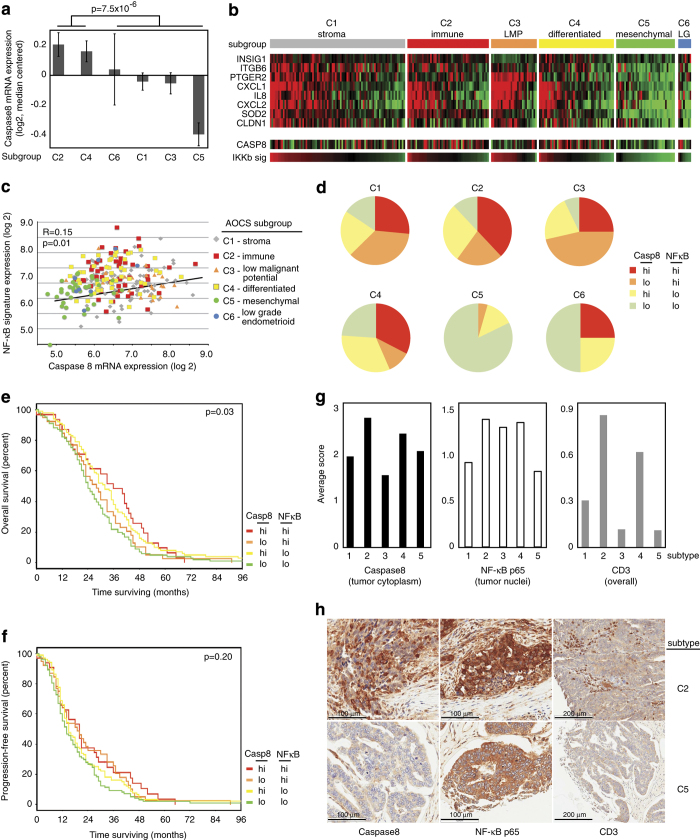
Caspase8 expression and NF-*κ*B expression are correlated in patient samples. (**a**) Caspase8 mRNA expression quantified in subtypes of ovarian cancer as defined by the AOCS (average of probe sets 207686_at and 213373_s_at from Geo Dataset GSE9899), is highest in the C2 and C4 subtypes (*P*=7.5×10^−6^, based on *t*-test between groups C2+C4 *versus* others). (**b**) Patient sample subgroups were ranked by average expression of NF-*κ*B signature. Caspase8 relative expression is shown for each patient sample, demonstrating highest expression in C2 and C4 subgroups, and lowest in C5. (**c**) Caspase8 expression correlates with NF-*κ*B gene signature expression in AOCS patient samples from subtypes C2 and C5 (*P*=0.0002). (**d**) High *versus* Low expression of either Caspase8 or NF-*κ*B gene signature was defined by median value across the entire data set. Shown are the proportions of samples within each subgroup with High (above the median) or Low (below the median) expression of either Caspase8 or NF-*κ*B gene signature. (**e**) Overall survival of patients in each Caspase8/NF-*κ*B category as defined in **d**. Patients with elevated expression of both Caspase8 and NF-*κ*B had the longest median overall survival (*P*=0.03). (**f**) Progression-free survival of patients in each Caspase8/NF-*κ*B category as defined in **d**. (**g**) Immunohistochemistry (IHC) average intensity scores of tumor cells expressing Caspase8, NF-*κ*B-p65 and CD3 protein levels in AOCS tissue microarrays containing representative subtypes (C1, *n*=17; C2, *n*=19; C3, *n*=9; C4, *n*=20; C5, *n*=19). Percent positive cells were scored as 0 (<10%), 1 (10–24%), 2 (25–50%), 3 (50–74%) or 4 (75–100%). Staining intensity was scored as 0 (neg), 1 (weak), 2 (moderate), 3 (positive). In addition, tumors were scored for nuclear and cytoplasmic stain according to the above rubric. A composite score was tabulated by multiplying intensity score by percent positive score, and reported as average for each of the AOCS subtypes in the tissue microarrays. (**h**) Representative images of patient tissue from C2 subtype, showing high CASPASE 8, nuclear NF-*κ*B-p65 and T-cell infiltrate, contrasted with C5 subtype showing comparatively low markers.

We sought to relate Caspase8 and NF-*κ*B in gene expression profiles from primary ovarian cancers, to investigate biologic and clinical relevance of the co-dependence uncovered during our shRNA screen. The AOCS contains gene expression profiles from 283 primary ovarian cancers.^4^ Six subtypes of ovarian cancer were identified, and termed C1–C6. Caspase8 expression was highest in the subtypes characterized by an immune signature (C2) and a differentiated signature (C4) (Figure 3a). Expression of our nine-gene ovarian cancer NF-*κ*B signature^9^ was prominent in C2 and C4 subtypes where Caspase8 was highest (Figure 3b). C3 subtype expressed high NF-*κ*B genes, with intermediate Caspase8 expression. Caspase8 expression was significantly correlated with our NF-*κ*B signature across the whole data set (Figure 3c, *P*=0.01) and even more significantly when analyzing only C2 and C5 subgroups (*P*=0.0002). In particular, immune and differentiated subtypes expressed both Caspase8 and NF-*κ*B signature above the median, whereas mesenchymal/C5 was more likely to have low expression of both Caspase8 and NF-*κ*B signature (Figure 3d). Co-expression of Caspase8 and NF-*κ*B signature suggested a functional dependence in immune and differentiated subgroups, supporting our findings of the co-dependence of IKK*β* and Caspase8 identified in the shRNA sensitization screen.

Expression of both Caspase8 and NF-*κ*B signature above the median was associated with the best overall survival (OS) in this data set (Figure 3e, *P*=0.03). Conversely, low expression of both resulted in the shortest OS. There was a similar trend in progression-free survival (PFS), without reaching statistical significance (Figure 3f, *P*=0.20). We sought to determine whether Caspase8 was expressed in the ovarian cancer cells themselves, especially in subtypes with immune cell infiltration. AOCS provided a tissue microarray containing 9–20 representative samples for each subtype, totaling 84 samples. Protein expression of Caspase8, NF-*κ*B-p65 and CD3 T-cell marker was assessed by IHC and blind-read by two independent pathologists. Immune/C2 and differentiated/C4 showed the highest cytoplasmic Caspase8 within tumor cells, consistent with gene expression results (Figures 3g and h). Nuclear p65 indicates active NF-*κ*B signaling. Higher frequency of nuclear NF-*κ*B-p65 was present in C2, C3 and C4 subtypes, whereas C5 had the lowest expression, again consistent with gene expression profiles. Nuclear p65 was elevated in subtypes that also had high expression of our previously published NF-*κ*B signature. This finding links the gene expression signature to the evidence of NF-*κ*B activity at the protein level, further supporting the gene signature as a marker of active NF-*κ*B signaling in ovarian cancers. Of note, Caspase8 and NF-*κ*B-p65 proteins were expressed by tumor cells themselves, suggesting that gene expression findings represent tumor cell pathways, and not immune infiltrate. Consistent with published findings,^4^ CD3-positive T-lymphocytes were notably higher in C2 and C4 subtypes. This supports a biologically relevant cooperative role between Caspase8 and NF-*κ*B, particularly in the immune/C2 and differentiated/C4 subtypes of ovarian cancer.

**Figure 4:**
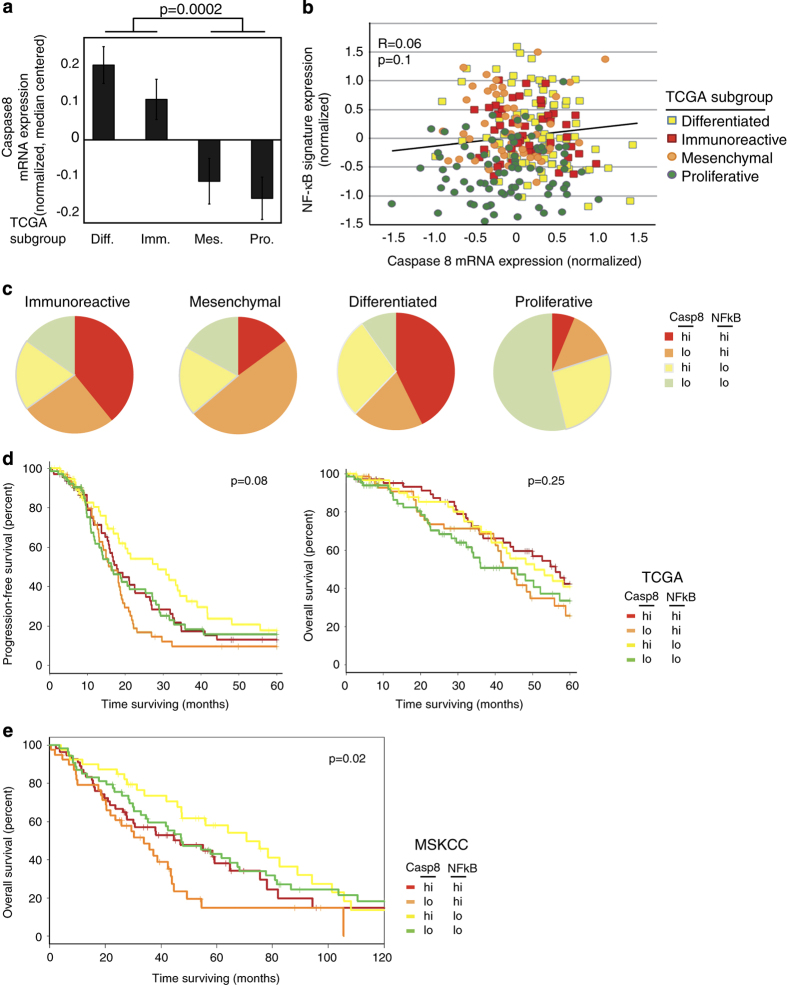
Caspase8 is differentially expressed and correlates with patient outcome. (**a**) Caspase8 mRNA expression quantified in subtypes of ovarian cancer as defined by TCGA is highest in the differentiated and immunoreactive subtypes (*P*=0.0002 based on *t*-test). (**b**) Caspase8 expression shows a strong trend towards correlation with NF-*κ*B gene signature expression across the TCGA data set (*P*=0.1) (**c**) High *versus* Low expression of either Caspase8 or NF-*κ*B gene signature was defined by median value across the entire data set. Shown are the proportions of samples within each subgroup with High (above the median) or Low (below the median) expression of either Caspase8 or NF-*κ*B gene signature. (**d**) Progression-free survival of patients in each Caspase8/NF-*κ*B category as defined in **c**. A strong trend was found between the categories, this time with high Caspase8 and low NF-*κ*B having the longest progression-free survival (*P*=0.08), but there was no significant correlation found in overall survival, where follow-up times were shorter. (**e**) Overall survival of patients in a third data set from MSKCC, divided into each Caspase8/NF-*κ*B category as defined in similar manner. Patients with elevated expression of Caspase8 but low NF-*κ*B had the longest median overall survival (*P*=0.02).

These findings were supported in TCGA, containing gene expression profiles from 489 primary ovarian cancers.^3^ Similar to AOCS, Caspase8 was highest in the immunoreactive and differentiated subtypes (Figure 4a). Caspase8 showed a positive relationship with NF-*κ*B genes in TCGA (Figure 4b, *P*=0.1). TCGA subtypes also exhibited a similar pattern of Caspase8 and NF-*κ*B: immunoreactive and differentiated subtypes co-expressed Caspase8 and NF-*κ*B signature in a higher percentage of cases, compared with the proliferative type where both Caspase8 and NF-*κ*B signature were low (Figure 4c). In survival analyses, the follow-up period was shorter than AOCS, and neither PFS nor OS showed significant subgroup differences within the TCGA data sets, but the trend in OS was similar to AOCS (Figure 4d). A third data set of gene expression in ovarian cancer, previously showing poor OS with high expression of the NF-*κ*B gene signature,^9,22^ was analyzed. In this MSKCC data set as well, higher Caspase8 was associated with longer OS when compared with low Caspase8, in the setting of either high (red compared with orange) or low (yellow compared with green) NF-*κ*B signature expression (Figure 4e). This prompted further consideration of the impact of Caspase8 expression on OS of women with ovarian cancer.

### Caspase8 is required for apoptosis upon TNF*α* stimulation and IKK*β* inhibition

**Figure 5:**
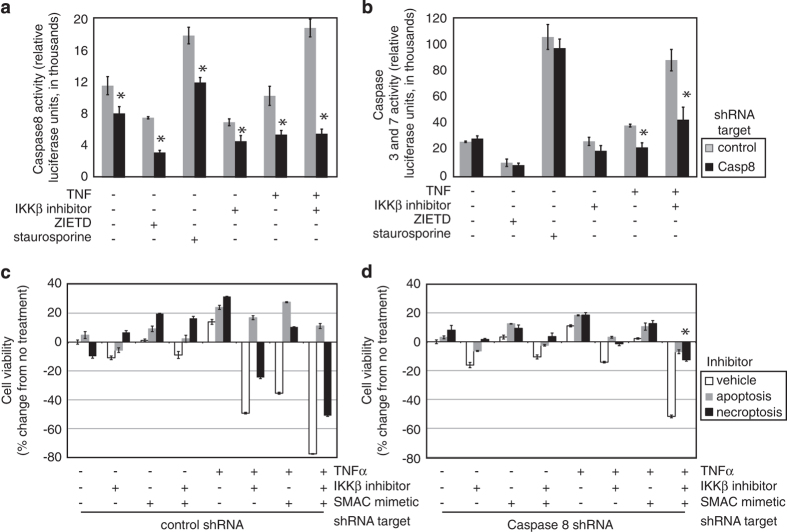
Necroptosis, but not apoptosis, is prominent in Caspase8-depleted cells. (**a**) Cells expressing either control or Caspase8 shRNA were exposed to Caspase8 inhibitor ZIETD (25 *μ*M), staurosporine (1 *μ*M), TNF*α* (10 ng/ml) and/or IKK*β* inhibitor (2.5 *μ*M), to induce caspase activity. Results are expressed as average luciferase units ±S.E.M., *n*=16. *P<0.05 based on *t*-test. (**b**) CASPASE 3 and 7 activity was measured in Ovcar3 cells exposed to similar conditions as in **a**. (**c**) Control shRNA-transduced Ovcar3 cells were exposed to IKK*β* inhibitor with or without TNF*α* stimulation, in the presence of apoptosis inhibitor ZIETD, or NEC1, a known inhibitor of necroptosis. Cells were treated for 18 h with TNF*α* (10 ng/ml), IKK*β* inhibitor (2.5 *μ*M), SMAC-mimetic (birinapant 200 nM), and/or ZIETD (25 *μ*M) or NEC1 (25 *μ*M) as indicated. Viability was assessed by XTT assay. Results are expressed as average±S.E.M., *n*=8. (**d**) Caspase8-depleted cells were treated as in **b**.

**Figure 6:**
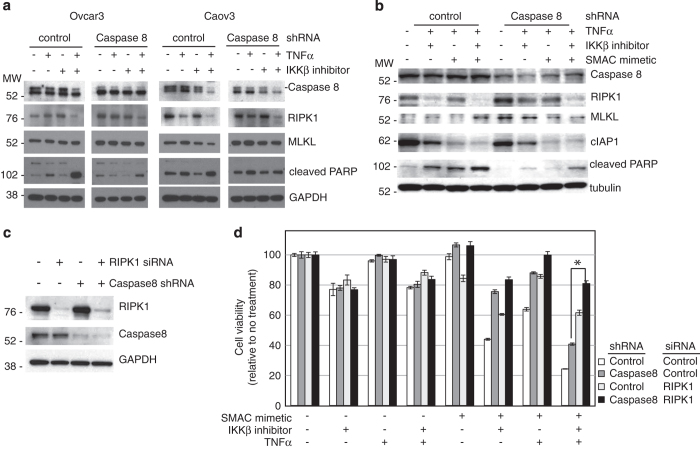
Caspase8-depleted ovarian cancer cells show evidence of cell death by necroptosis. (**a**) Western analysis was performed on cell lysates obtained from Ovcar3 and Caov3 cells expressing control or Caspase8 shRNA #2, after treatment with IKK*β* inhibitor (2.5 *μ*M) and/or TNF*α* (10 ng/ml) for 18 h. Protein levels of Caspase8, RIPK1 (uncleaved, 78 kDa), MLKL and cleaved PARP are shown. GAPDH was used as loading control (**b**) Western analysis was performed on cell lysates obtained from Ovcar3 cells expressing control or Caspase8 shRNA #2, after treatment with IKK*β* inhibitor (2.5 *μ*M), SMAC-mimetic (birinapant 200 nM) and/or TNF*α* (10 ng/ml) for 18 h. Protein levels of Caspase8, RIPK1 (uncleaved, 78 kDa), MLKL, cIAP1 and cleaved PARP are shown (upper). *β*-Tubulin was used as loading control. (**c**) Ovcar3 cells expressing either control or Caspase8 shRNA #2 were transiently transduced with control or RIPK1 siRNA. (**d**) Ovcar3 cells described in **c** were exposed to IKK*β* inhibitor with or without TNF*α* stimulation, in the presence of apoptosis inhibitor (ZIETD) or necroptosis inhibitor (NEC1). Cells were treated for 18 h with TNF*α* (10 ng/ml), IKK*β* inhibitor (2.5 *μ*M), SMAC-mimetic (birinapant 200 nM) and/or ZIETD (25 *μ*M) or NEC1 (25 *μ*M) as indicated. Viability was assessed by XTT assay. Results are expressed as average±S.E.M., *n*=8, *P<0.01 based on *t*-test, and shown as percent change from no treatment control.

TNF*α* can promote cell proliferation or apoptosis.^23^ We confirmed that Caspase8 mediated extrinsic apoptosis with short-term exposure to TNF*α*. Of note, this situation differs from that in the shRNA screen, which was performed over 10–14 days, without TNF*α*. In this short-term assay, Ovcar3 cells expressing Caspase8 shRNA or control were treated with IKK*β* inhibitor, TNF*α* or the combination (Figure 5a). Ovcar3 basal Caspase8 activity was decreased by ZIETD (a known inhibitor of Caspase8) or IKK*β* inhibitor, but increased by staurosporine (positive control). TNF*α* stimulation alone did not significantly affect Caspase8 activity, but combined TNF*α*/IKK*β* inhibitor prominently increased Caspase8 activity in control cells, comparable to staurosporine. In Caspase8-depleted cells, as expected, Caspase8 was uniformly less active, showing the largest difference in cells treated with TNF*α* and IKK*β* inhibitor, which activates extrinsic apoptosis (Figure 5a, *P*<0.05). Caspase8 activity propagates extrinsic apoptosis, leading to Caspase3 activation in the common apoptosis pathway.^24^ Caspase3 activity followed a similar pattern, except that Caspase8 depletion had little effect on staurosporine-induced (intrinsic pathway) Caspase3 activation (Figure 5b). Importantly, Caspase8 depletion significantly attenuated Caspase3 activity downstream of TNF*α*, especially when IKK*β* was inhibited (Figure 6b, *P*<0.05). This short-term apoptosis activity is clearly different from the long-term cell death that occurred during the shRNA library screen, in the absence of TNF*α*.

We proceeded to ask whether inhibition of necroptosis would prevent cell death in Caspase8-depleted cells. Cells transduced with control shRNA were stimulated to proliferate with TNF*α* alone. TNF*α* and IKK*β* inhibitor induced 49% cell death (Figure 5c). Similarly, TNF*α* with birinapant produced 35% cell death. Co-treatment with IKK*β* inhibitor and birinapant, in the presence of TNF*α*, increased cell death (to 78%). Of note, ovarian cancer cell lines, when co-treated with birinapant and TNF*α* exhibited >50% cell death at clinically achievable doses of birinapant^25^ (Noonan *et al*., manuscript in preparation). Interestingly, cells with control shRNA appeared to die mostly by apoptosis under these conditions, as evidenced by the fully protective effect of apoptosis inhibitor ZIETD, but not by NEC1, a specific inhibitor of RIPK1 and necroptosis.^26^ Caspase8-depleted cells were less proliferative with TNF*α* stimulation, and less susceptible to short-term killing with TNF*α*, IKK*β* inhibitor and birinapant, underscoring the dual role for Caspase8 in these cells (Figure 5d, *P*<0.05). Consistent with our hypothesis, however, Caspase8-depleted cells were almost completely rescued by NEC1, significantly more than cells with control shRNA. These data suggest a greater role of necroptosis when Caspase8 is suppressed.

### RIPK1 mediates necroptotic cell death when Caspase8 is deficient

RIPK1 is critical following TNFR1 engagement in lymphocytes,^13^ and promotes NF-*κ*B signaling with p43-cFLIP and cIAP1. In concert with IAPs, RIPK1 down-modulates the availability of Caspase8 for apoptosis. Without cIAP1, RIPK1 is cleaved by Caspase8; alternatively, uncleaved RIPK1 causes Caspase8-independent necroptosis.^27^ Although Caspase8 promotes apoptosis, its activity also inhibits necroptosis. Thus, we tested whether loss of Caspase8, while diminishing apoptosis, would effectively predispose to necroptosis, owing to increased availability of RIPK1. This mechanism could be responsible for the increased cell death detected upon Caspase8 depletion and IKK*β* inhibitor in the shRNA screen. Suppression of cIAP1 with birinapant should additionally enhance the combined effect of Caspase8 depletion and IKK*β* inhibitor under TNF*α* stimulation.^28^

Changes in RIPK1 and related pathway proteins were analyzed in Ovcar3 and Caov3 cells exposed to TNF*α*, IKK*β* inhibitor (Figure 6a) and/or birinapant (Figure 6b) to understand the downstream mechanisms by which IKK*β* inhibitor, coupled with Caspase8 depletion, led to cell death in our sensitization screen. Without TNF*α*, Caspase8 depletion stabilized full-length RIPK1 (Figures 6a and b). RIPK1 cleavage by Caspase8 is required for apoptosis in some lymphoid cell lines.^29^ Our data showing stabilization of RIPK1 are consistent with the idea that Caspase8 knockdown predisposed ovarian cancer cells to non-apoptotic cell death. Caspase8 depletion also increased mixed lineage kinase domain-like (MLKL), a protein associated with necroptosis.^30–32^ Birinapant increased MLKL in the absence of Caspase8 (Figure 6b). Conversely, cIAP1 levels diminished following IKK*β-*inhibitor and birinapant exposure, consistent with decreased protection from cell death. Stabilization of RIPK1 and rise in MLKL, in the setting of decreased caspase activation and decreased PARP cleavage, suggest that IKK*β*-inhibited, Caspase8-depleted ovarian cancer cells die by necroptosis, a RIPK1-dependent, Caspase8-independent form of programmed cell death.

To confirm that RIPK1 inhibition rescued Caspase8-depleted cells, we knocked down RIPK1 using siRNA instead of chemical inhibition with NEC1. RIPK1 knockdown was efficient in cells stably transfected with shRNA against control or Caspase8 (Figure 6c). Cells with Caspase8 knockdown or control shRNA, transfected with RIPK1 siRNA or control, were exposed to TNF*α*, IKK*β* inhibitor and/or birinapant. Caspase8-depleted cells were again almost completely rescued (80% viability) by RIPK1 knockdown (Figure 6d, *P*<0.05). Taken together, these data support our hypothesis that Caspase8 depletion in combination with IKK*β* and IAP inhibition both disrupts TNF*α*/IKK*β*−IKK IAP inhi*κ*B pro-survival effects and promotes RIPK1-dependent necroptosis.

## Discussion

A pro-survival role for Caspase8 is not previously described in ovarian cancer. Our findings in patient samples suggest a subtype-specific role for Caspase8 in ovarian cancer, where the activity of NF-*κ*B and expression of Caspase8 was coordinated specifically in the immune-related subtypes of ovarian cancer. Importantly, low Caspase8 activity was associated with worse outcome in women with newly diagnosed ovarian cancer. *In vitro* experiments showed poor ability to initiate apoptosis without Caspase8, but improved killing with additional necroptosis when SMAC-mimetic birinapant was added to the inhibition of NF-*κ*B signaling. Thus, low Caspase8 could identify patients who may benefit from therapies designed to bypass apoptosis and induce necroptosis.

We describe a dual role for Caspase8 in ovarian cancer. Our previous studies identified ovarian cancers with active NF-*κ*B, and sensitivity to IKK*β* inhibitor. In this study, we aimed to increase the anticancer effect of IKK*β* inhibitor via selective identification of shRNAs that decreased cell viability in the presence of IK*κβ* inhibitor but ignored shRNAs that were toxic in a manner not additive with IK*κβ* inhibitor.^33^ Caspase8 emerged as a cooperating gene, and validated in shRNA knockdown experiments. Caspase8 propagates apoptosis, and so this result was initially counterintuitive. Caspase8, however, also has known involvement with NF-*κ*B signaling.^29,34^ For example, when Caspase8 heterodimerizes with p43-cFLIP, its autocleavage is inhibited. The dimer associates with RIPK1, preventing necroptosis and activating NF-*κ*B.^35^ In the absence of Caspase8, we show that this mechanism of NF-*κ*B pathway activity is interrupted. This mechanism is not well described in epithelial cancers such as ovarian cancer.

NF-*κ*B inhibition induces apoptosis in lymphomas.^36^ In the current study, Ovcar3 cells required additional triggers to undergo rapid cell death. Under such conditions, Ovcar3 cells underwent apoptosis when Caspase8 was present, but necroptosis when Caspase8 was low and RIPK1 stabilized. These results parallel, but do not overlap, recent findings in multiple myeloma, where Caspase10 was the critical molecule, protecting myeloma cells from autophagy rather than necroptosis.^37^

Many epithelial cancers, including ovarian cancer, develop resistance to therapies designed to induce apoptosis.^38^ Our studies indicate that Caspase8 poises cells towards apoptosis triggered by extrinsic stimuli such as TNF*α*, but NF-*κ*B inhibits this process. Cells with low Caspase8, however, resisted apoptosis. In these cells, death was dependent on RIPK1, pointing to necroptosis as a potential avenue to overcome resistance to apoptosis in cancer cells, and improve outcome in women whose ovarian cancers express endogenously low Caspase8.

The clinically relevant SMAC-mimetic, birinapant further enhanced death of Caspase8-deficient cells. Phase 1 studies with SMAC-mimetics demonstrated little clinical activity as single agents, but combination therapy improved clinical benefit.^39,40^ In the preclinical setting, SMAC-mimetic plus TRAIL agonist enhanced signaling through death receptors DR4 and DR5,^41,42^ but this effect would require active Caspase8. Proteasome inhibition may also enhance killing by SMAC-mimetics, by stabilizing SMAC and cleaved Caspase3 or reducing cIAP1 expression.^43–45^ Our results suggest that blocking NF-*κ*B signaling, one specific effect of proteasome inhibitors, is critical for SMAC-mimetic induction of cell death.

Taken together, our studies demonstrated that interference with NF-*κ*B-driven survival effects could be advantageous in promoting apoptotic or necroptotic cell death in ovarian cancers. This could lead to biomarker-driven therapeutic strategies to increase the effectiveness of standard chemotherapy. Such a strategy may be particularly important to women with Caspase8-deficient ovarian cancer, and cancers resistant to chemotherapies designed primarily to induce apoptosis.

## Materials and Methods

### RNA interference library screen

The ovarian serous adenocarcinoma cell line Ovcar3 was infected with a bar-coded retroviral shRNAs library packaged with the amphotropic receptor in CeB cells, as described.^33,46,47^ Infected cells underwent 4 days selection with 2 *μ*g/ml puromycin (Sigma-Aldrich, St. Louis, MO, USA) to allow constitutive shRNA expression before treatment with 2.5 *μ*M IKK*β* inhibitor IV (EMD Biosciences, Billerica, MA, USA). Control cells were treated with equal volumes of DMSO. Cells were cultured continuously under puromycin selection and inhibitor or control for 10 or 14 days in two independent experiments. Cells were collected, genomic DNA was isolated and the unique 60-base pair molecular barcode in each shRNA vector was amplified by PCR. High-throughput Illumina sequencing of barcodes compared the relative abundance of each shRNA in the IKK*β* inhibitor-treated and control cells. Four biological replicates were performed; statistical analysis identified shRNAs significantly depleted or enriched in IKK*β* inhibitor-treated cells *versus* control according to previously established parameters.^33,46,47^ Briefly, the fold change owing to shRNA effect and standard error thereof were calculated with logistic regression to estimate relative probability that an shRNA read came from an IKK*β* inhibitor-treated *versus* untreated experiment. This model included a normalization factor that was constant for all genes from a given shRNA pool in a given experiment, including normal random effects representing gene by experiment interactions.

### Analysis of individual shRNAs from library

Using primers designed for the shRNA library (Supplementary Table 2), four Caspase8 shRNAs #1(oligo 888), #2 (oligo1512), #3(oligo1583), #4(oligo2172) and a control scrambled shRNA were cloned into pRSMX retroviral vectors as described before^9^ and Ovcar3 cells were transduced independently with each clone. Cells were collected at days 4, 7 and 10 after selection and total RNA used for qPCR analysis. Protein expression was analyzed by standard western blot methods and mRNA expression was analyzed by quantitative real-time PCR. Total RNA was obtained from cells using Trizol (Ambion, Lafayette, CO, USA), and cDNA was synthesized using Superscript II RT (Invitrogen, Carlsbad, CA, USA). PCR was performed using Taqman PCR Master Mix (Applied Biosystems, Foster City, CA, USA) on an ABI 7900HT thermal cycler. B2M expression was used as an internal control to normalize between samples. Primer probe sets for Caspase8 (Hs01018151_m1) and B2M control (4333766F, Applied Biosystems).

### Cell viability assays

Ovcar3, Ovcar5, Igrov1 and Ovcar8 cells were a gift from Dr. Elise Kohn, and previously described^9^ Caov3 cells were from ATCC (American Type Culture Collection, Manassas, VA, USA) and PEO1 cells were a generous gift of Dr. James Brenton (University of Cambridge, UK). All ovarian cell lines were authenticated by short tandem repeat analysis performed by the Molecular Detection Group, SAIC, Frederick National Laboratory, in reference to the ATCC profile for the same cell line (www.atcc.org). Adherent ovarian cancer cell viability was assessed using XTT (Sigma-Aldrich) as described.^48^ Briefly, cells were seeded in 96-well plates at a density of 1–2000 cells/50 *μ*l/well and incubated^9^ for 24 h. To analyze the sensitizing effect of Caspase8 knockdown on the lethality of IKK*β* inhibition observed in the shRNA library experiments, ovarian cancer cells were Caspase8 depleted using each of four shRNA clones or control scrambled shRNA. For shRNA library viability studies, after selection, cells were divided into two populations, seeded into 96-well plates as described and treated either with vehicle or with 2.5 *μ*M IKK*β* inhibitor IV for 10 days. Drugs were prepared as DMSO stocks and serially diluted in 10% RPMI medium to 2× concentrations, then added in 50 *μ*l aliquots to each cell well. Final DMSO concentrations did not exceed 0.5%, previously established to be nontoxic to the cells. Plates were incubated for up to 10 days, and inhibitors replenished every 3–4 days. Cell viability was assessed by incubating cultures with XTT^48^ and absorbances read in a Spectramax M5 plate reader (Molecular Devices, Downington, PA, USA). Cell density in treated wells was expressed as a percent of vehicle-treated control wells. Experiments included 16 replicate samples per point and were repeated at least three times. All viability studies were performed according to the above format, using either TNF*α* 10 ng/ml, 25 *μ*M Caspase8 inhibitor ZIETD (Z-IETD-FMK, FMK-007, R&D Systems, Minneapolis, MN, USA), 25 *μ*M Q-AEVD-OPH (OPH033, MP Biomedicals, Solon, OH, USA, Caspase10 inhibitor), 25 *μ*M Q-VD-OPH (OPH109, MP Biomedicals, broad-spectrum caspase inhibitor), 20 *μ*M NEC1 (Necrostatin-1, Tocris, Ellisville, MI, USA) or birinapant (Tetralogic Pharmaceuticals, Malvern, PA, USA) added to cells either alone or in combination with 2.5 *μ*M IKK*β* inhibitor IV (EMD Biosciences), or as described.

### NF-*κ*B activity reporter assay

Ovcar3 cells were transduced with a lentiviral vector containing an NF-*κ*B transcriptional regulatory element, using the Cignal Lenti Reporter System (CLS-013L, Qiagen, Valencia, CA, USA). According to manufacturer’s specifications, cells were selected and established as a stable pathway sensor Ovcar3 subline. Briefly, cells were plated in 96-well plates at a density of 10 000 cells/well. After overnight attachment, cells were exposed to serum starvation medium containing 0.5% FBS for 24 h. Inhibitors were added for 1 h after which TNF*α* (300-01A, PeproTech, Rocky Hill, NJ, USA) was added to stimulate NF-*κ*B activity for 18 h. Control wells received vehicle alone. Luciferase activity was measured using the Luciferase Assay System (E4030, Promega, Madison, WI, USA) according to manufacturer’s instructions, and a Spectramax M5 plate reader (Molecular Devices). Luciferase units were normalized to viable cell number, obtained by XTT assay, on duplicate assay plates. Alternatively, Ovcar3 cells subjected to scrambled or Caspase8 shRNA knockdown were first selected with puromycin as described above in Materials and Methods. After selection, cells were transduced with a lentiviral vector containing an NF-*κ*B transcriptional regulatory element, using the Cignal Lenti Reporter System (CLS-013L), according to manufacturer’s specifications and allowed 72 h for maximum vector expression. Transient reporter assays were subsequently performed for 18 h as described above in this section.

### Quantitative PCR assessment of NF-*κ*B target gene expression

One microgram of total RNA obtained with Trizol as described above was converted to cDNA using iScript cDNA Synthesis Kit (Bio-Rad, Hercules, CA, USA, 170-8890) in a 20 *μ*l reaction. cDNA was then diluted to 1 : 5 in H_2_O and 2 *μ*l was used in each 20 *μ*l real-time PCR reaction (QuantiTect SYBR Green PCR Kit, Qiagen, 204143). Quantification was performed in triplicates by ViiA7 Real-Time PCR System (Applied Biosystems). Each mRNA expression level was normalized by that of GAPDH. The QuantiTect primers CFLAR/cFLIP (QT00064554), CXCL1 (QT00199752), CXCL2, (QT00013104), CXCL8/IL-8 (QT00000322), CASP8 (QT00052416), CLDN1 (QT00225764) and GAPDH (QT00079247) were purchased from Qiagen.

### Caspase activity assays

Caspase8 and Caspase3 activity was measured using freshly harvested cell pellets after 18 h treatments. Activity data were normalized to viable cell number. Control and Caspase8 knockdown Ovcar3 cells were analyzed by cell-based Caspase8 and Caspase3/7 (G8200, G8090) luminescence assays (Promega) after 18 h treatment, according to manufacturer’s specifications.

### siRNA studies

ON-TARGET plus human RIPK1 (Cat. #L-004445) and negative siRNAs were purchased from Thermo Scientific (Lafayette, CO, USA). Ovcar3 cells previously transduced with control or Caspase8 shRNA #2 were seeded at a density of 1×10^5^/well in a six-well plate one day before siRNA transfection. siRNAs were transiently transfected using DharmaFECT1 at a final concentration of 20 nM. RIPK1 knockdown was assessed at 48 h post transfection. Thirty micrograms of total lysate was loaded on gels for western blot analysis, performed as described below.

### Western analysis

Total protein was extracted from cell cultures using RIPA buffer (sc-24948) according to manufacturer protocol (Santa Cruz Biotechnology, Dallas, TX, USA) and concentrations estimated with the BCA Protein Assay Kit (Thermo Scientific). SDS-PAGE and western analysis were performed using, respectively, the NuPage system (Invitrogen) and the Supersignal Chemiluminescent Substrate system (Thermo Scientific). The following antibodies were used: cIAP1 (AF8181, R&D Systems), Caspase8 (90A992, Thermo Scientific and 9746, Cell Signaling Technology, Boston, MA, USA), cFLIP (ab8421, Abcam, Cambridge, UK), RIP1 (3493, Cell Signaling Technology) and cleaved PARP (9541, Cell Signaling Technology), MLKL (sc-130172, Santa Cruz Biotechnology), GAPDH (MAB374, Millipore, Billerica, MA, USA) and *β*-tubulin (T5201, Sigma-Aldrich).

### Patient data sets

Geo Dataset 9899 contains the gene expression for the AOCS samples.^4^ Data were log2 transformed before calculation. The TCGA data portal is found at https://tcga-data.nci.nih.gov/docs/publications/ov_2011/.^3^ Expression data in the portal have been normalized to noncancer controls. Gene expression data were grouped into designated subtypes, as per the respective publications, and the average expression of Caspase8 and NF-*κ*B gene signature was calculated. The NF-*κ*B gene signature is previously published.^9^ Pearson correlation (*R*) of the two averages across patient samples was calculated using Microsoft Excel, and the *P*-value was calculated using online calculator http://www.danielsoper.com/statcalc3/calc.aspx?id=44. Kaplan–Meier survival plots were generated using online calculator at https://statcom.dk/K-M_plot.

### Immunohistochemistry

Ovarian cancer subtype-specific tissue microarrays were obtained through the Australian Ovarian Cancer Study, under Materials Transfer Agreement, and are exempt from institutional Human Subjects Protection by internal review (NIH Office of Human Subjects Research, Exemption #11948). Immunohistochemistry was performed on formalin-fixed, paraffin- embedded tissue arrays prepared from 1 mm representative cores using standard technique by the NCI-Frederick Histotechnology Laboratory and optimized concentrations of Caspase8 (90A992, Thermo Scientific), NF-*κ*B-p65 (sc-372, Santa Cruz Biotechnology) and CD3 (MCA1477, Serotec, Raleigh, NC, USA) antibodies. Pathology evaluation was performed by a combination of the following semiquantitative methods: percent positive cells were scored as 0 (<10%), 1 (10–24%), 2 (25–50%), 3 (50–74%) and 4(75–100%). Staining intensity was scored as 0 (neg), 1 (weak), 2 (moderate), 3 (positive). In addition, tumors were scored for nuclear and cytoplasmic stain according to the above rubric. Scores were tabulated and reported as average for each of the AOCS subtypes in the tissue microarrays. Pathology evaluation was independently blind-read by two pathologists.

Cell pellet arrays were prepared by collecting 0.5–1 million cells without enzymatic treatment; monolayers were rinsed in warm medium and dislodged with a cell scraper before pelleting. Drained pellets were treated in ice 2–5 min with 8 *µ*l of 1 U/*µ*l Thrombin (Sigma), followed by 5 *µ*l fibrinogen 10 mg/ml 2–5 min at room temperature. Clotted cells were pelleted, supernatant discarded and pellets fixed in 4% buffered paraformaldehyde for 16 h at room temperature. Cells were pelleted again, fixative discarded and pellet washed and kept in 70% ETOH 24–48 h before paraffin embedding. Cell pellet microarray slides were stained by standard technique in NCI-Frederick Histotechnology Laboratory using the above-described Caspase8 and NF-*κ*B-p65 antibodies. Nuclear high-intensity staining was scored by tabulating average counts of three to four exclusive areas per pellet.

## Abbreviations

NF-*κ*B: nuclear factor kappa B
TNF*α*: tumor necrosis factor-alpha
IKK*β*: IKappaB Kinase, IKBKB, IKK-2
SMAC: small mitochondrial activator of caspases
IAP: inhibitor of apoptosis proteins
TCGA: Cancer Genome Atlas
AOCS: Australian Ovarian Cancer Study
MSKCC: Memorial Sloan Kettering Cancer Center
C-FLIP: Caspase8 and FADD-like apoptosis regulator
TRAF2: TNF receptor-associated factor 2
cIAP1: baculoviral IAP repeat-containing protein 1
RIPK1: receptor-interacting serine/threonine-protein kinase 1
MLKL: mixed lineage kinase domain-like
GAPDH: anti-glyceraldehyde-3-phosphate dehydrogenase
shRNA: short-hairpin RNA.
