# Supplementary material for: A dual role for Caspase8 and NF-κB interactions in regulating apoptosis and necroptosis of ovarian cancer, with correlation to patient survival
Source: Cell Death Discov. 2015 Dec 14;1:15053–. doi: 10.1038/cddiscovery.2015.53 (PMC5198842; doi:10.1038/cddiscovery.2015.53)
Supplement: Supplementary Information [file cddiscovery201553-s1.doc]

**Description of supplementary data files**

**Supplementary data file 1 (Microsoft Word)** is Table S1: shRNAs cytotoxic in a synergistic manner with IKKβ inhibition. shRNA library used in screen contained twelve shRNAs for most represented genes. Cytotoxic shRNAs to IKKβ-inhibited Ovcar3 cells were identified as described in Methods. This table lists 30 genes that were toxic to Ovcar3 cells in a synergistic manner with IKKβ inhibition at day 10, and 15 genes at day 14 (fold difference >0.6). Genes listed contained a minimum of 3 significantly toxic shRNAs (p<0.05). The CASPASE 8 gene set was highly significant, with 4 common toxic shRNAs in two experiments.

**Supplementary data file 2 (Microsoft Word)** is Table S2: Library primers used for shRNAs into pRSMX vectors for transduction of Ovcar3 cells.

**Supplementary data file 3 (EPS)** is Figure S1: NF-κB signaling and cell proliferation are correlated in ovarian cancer cells. (A) NF-κB signaling in Ovcar3 cells was reduced in a dose-dependent manner by IKKβ inhibitor treatment. IC50 inhibitor concentration suppressed signaling by 75% after 18h of treatment. Data are arbitrary luciferase units normalized to XTT, n=8 replicates at each concentration of IKKβ inhibitor. (B) Ovcar3 cells were exposed to varying IKKβ inhibitor IV concentrations over 3 days or 7 days in culture and viability was measured by XTT assay. IKKβ inhibition reduced ovarian cancer cell proliferation *in vitro* over 7 days in culture, with an IC50 concentration of 2.5 µM. Data are XTT units percent control, n=4 replicates at each concentration of IKKβ inhibitor. Error bars represent S.E.M.

**Supplementary data file 4 (EPS)** is Figure S2: Caspase8 shRNAs inhibit gene expression and protein levels in ovarian cancer cells. Ovcar3 cells were transduced with selected Caspase8 shRNAs from shRNA library as described in Supplementary Table 1. (A) Caspase8 mRNA expression in cells expressing shRNAs for 4, 7 and 10 days were assessed by real-time PCR. Data are percent control shRNA +/- S.E.M., n=3. (B) Western analysis of Caspase8 protein expression in Ovcar3 cells transduced with shRNAs described, after 4 days or 10 days.

**Supplementary data file 5 (EPS)** is Figure S3: Inhibition of caspase activity does not affect ovarian cancer cell viability. Inhibition of caspase activity does not affect ovarian cancer cell viability. Ovcar3 cells were exposed to Caspase8 inhibitor or pan-caspase inhibitor, or to combined Caspase8 and IKKβ inhibitor for 10 days. Inhibitors were replenished every 3 days, and viability was measured by XTT. Results shown are average of 8 replicates, +/- S.E.M.
