# Supplementary material for: A dual role for Caspase8 and NF-κB interactions in regulating apoptosis and necroptosis of ovarian cancer, with correlation to patient survival
Source: Cell Death Discov. 2015 Dec 14;1:15053–. doi: 10.1038/cddiscovery.2015.53 (PMC5198842; doi:10.1038/cddiscovery.2015.53)
Supplement: Supplementary Table S1 [file cddiscovery201553-s2.doc]

| **SUPPLEMENTARY TABLE 1:** | |  |  |  |  | |
| --- | --- | --- | --- | --- | --- | --- |
| **DAY 10 - shRNA with significant difference between IKKβ inhibitor and control (p-value <0.05; Log fold >0.6)** | | | | | |  |
|  | Refseq starting bp for shRNA | Gene symbol | Log_2_ fold | p-value |  | |
|  | NM_000061_bp2522_L | BTK | 1.995959 | 0.001076688 |  | |
|  | NM_000061_bp2281_L | BTK | 0.6089388 | 7.93E-04 |  | |
|  | NM_000061_bp2346_L | BTK | 0.7250267 | 0.008502802 |  | |
|  |  |  |  |  |  | |
|  | NM_020439_bp2164_L | CAMK1G | 6.165544 | 0.01932593 |  | |
|  | NM_020439_bp2200_L | CAMK1G | 1.687428 | 0.01327476 |  | |
|  | NM_020439_bp489_L | CAMK1G | 1.021136 | 0.01862657 |  | |
|  |  |  |  |  |  | |
|  | NM_001080124_bp1322_L | CASP8 | 1.230866 | 3.83E-37 |  | |
|  | NM_001080124_bp1512_L | CASP8 | 0.7230204 | 3.63E-06 |  | |
|  | NM_001080124_bp888_L | CASP8 | 0.8300636 | 0.01570203 |  | |
|  | NM_001080124_bp2172_L | CASP8 | 0.6203021 | 0.01198977 |  | |
|  | NM_001080124_bp1583_L | CASP8 | 0.6646349 | 0.02924378 |  | |
|  |  |  |  |  |  | |
|  | NM_015076_bp439_L | CDC2L6 | 2.827438 | 1.42E-12 |  | |
|  | NM_015076_bp4084_L | CDC2L6 | 1.504062 | 1.20E-10 |  | |
|  | NM_015076_bp995_L | CDC2L6 | 1.432958 | 8.32E-07 |  | |
|  |  |  |  |  |  | |
|  | NM_001006658_bp2045_L | CR2 | 1.92188 | 1.41E-05 |  | |
|  | NM_001006658_bp189_L | CR2 | 1.299894 | 0.03304039 |  | |
|  | NM_001006658_bp1743_L | CR2 | 0.7472591 | 0.02787743 |  | |
|  |  |  |  |  |  | |
|  | NM_001319_bp1474_L | CSNK1G2 | 1.054501 | 1.10E-05 |  | |
|  | NM_001319_bp1691_L | CSNK1G2 | 0.6881255 | 0.008001526 |  | |
|  | NM_001319_bp2006_L | CSNK1G2 | 0.9473834 | 0.04462295 |  | |
|  |  |  |  |  |  | |
|  | NM_030636_bp878_L | EEPD1 | 0.8464715 | 0.006424749 |  | |
|  | NM_030636_bp3261_L | EEPD1 | 1.962846 | 0.02947291 |  | |
|  | NM_030636_bp2053_L | EEPD1 | 1.117155 | 0.03381183 |  | |
|  |  |  |  |  |  | |
|  | NM_005233_bp2997_L | EPHA3 | 2.893548 | 1.03E-07 |  | |
|  | NM_005233_bp4451_L | EPHA3 | 0.8262285 | 4.45E-14 |  | |
|  | NM_005233_bp2077_L | EPHA3 | 0.646289 | 0.004484747 |  | |
|  |  |  |  |  |  | |
|  | NM_004443_bp2550_L | EPHB3 | 0.7382504 | 0.007190981 |  | |
|  | NM_004443_bp2198_L | EPHB3 | 1.569568 | 0.02557222 |  | |
|  | NM_004443_bp3709_L | EPHB3 | 1.591359 | 0.04191356 |  | |
|  |  |  |  |  |  | |
|  | NM_001982.2_bp2989_L | ERBB3 | 1.490365 | 5.01E-05 |  | |
|  | NM_001982.2_bp584_L | ERBB3 | 1.974045 | 0.02984555 |  | |
|  | NM_001982.2_bp273_L | ERBB3 | 0.668619 | 0.02537105 |  | |
|  | NM_001982.2_bp2195_L | ERBB3 | 1.209132 | 0.03557663 |  | |
|  |  |  |  |  |  | |
|  | NM_016337_bp539_L | EVL | 1.67324 | 0.01770181 |  | |
|  | NM_016337_bp1349_L | EVL | 1.037183 | 0.01231955 |  | |
|  | NM_016337_bp1636_L | EVL | 1.181046 | 0.03172165 |  | |
|  |  |  |  |  |  | |
|  | NM_004119_bp2579_L | FLT3 | 1.577593 | 3.78E-08 |  | |
|  | NM_004119_bp925_L | FLT3 | 1.309309 | 1.00E-11 |  | |
|  | NM_004119_bp2475_L | FLT3 | 1.770363 | 0.002381566 |  | |
|  |  |  |  |  |  | |
|  | NM_006060_bp328_L | IKZF1 | 1.286896 | 5.22E-10 |  | |
|  | NM_006060_bp1677_L | IKZF1 | 1.056693 | 0.03147511 |  | |
|  | NM_006060_bp916_L | IKZF1 | 1.180427 | 0.03604983 |  | |
|  |  |  |  |  |  | |
|  | NM_000208_bp599_L | INSR | 0.9482069 | 2.78E-08 |  | |
|  | NM_000208_bp3916_L | INSR | 1.896021 | 0.002773977 |  | |
|  | NM_000208_bp3497_L | INSR | 0.9976293 | 0.01843493 |  | |
|  |  |  |  |  |  | |
|  | NM_152230_bp1615_L | IPMK | 2.26741 | 1.41E-08 |  | |
|  | NM_152230_bp1628_L | IPMK | 0.944139 | 5.11E-04 |  | |
|  | NM_152230_bp485_L | IPMK | 0.7253804 | 0.03081068 |  | |
|  |  |  |  |  |  | |
|  | NM_001098627_bp2023_L | IRF5 | 0.7841942 | 3.24E-04 |  | |
|  | NM_001098627_bp2060_L | IRF5 | 2.030034 | 0.001475763 |  | |
|  | NM_001098627_bp1409_L | IRF5 | 0.8318125 | 0.02607507 |  | |
|  |  |  |  |  |  | |
|  | NM_001024660_bp10572_L | KALRN | 1.520179 | 1.00E-04 |  | |
|  | NM_001024660_bp9072_L | KALRN | 1.279043 | 0.001121096 |  | |
|  | NM_001024660_bp8178_L | KALRN | 1.181956 | 0.003026421 |  | |
|  | NM_001024660_bp8959_L | KALRN | 0.9467801 | 0.008547358 |  | |
|  |  |  |  |  |  | |
|  | NM_002314_bp2497_L | LIMK1 | 2.189905 | 1.93E-10 |  | |
|  | NM_002314_bp2866_L | LIMK1 | 0.9347711 | 4.90E-04 |  | |
|  | NM_002314_bp2089_L | LIMK1 | 1.603219 | 0.003173285 |  | |
|  | NM_002314_bp1731_L | LIMK1 | 1.34758 | 0.004176859 |  | |
|  |  |  |  |  |  | |
|  | NM_014757_bp1757_L | MAML1 | 1.158769 | 1.69E-06 |  | |
|  | NM_014757_bp3296_L | MAML1 | 0.6515189 | 6.03E-06 |  | |
|  | NM_014757_bp5556_L | MAML1 | 0.6579938 | 0.007678872 |  | |
|  |  |  |  |  |  | |
|  | NM_139033.2_bp2807_L | MAPK7 | 3.138645 | 2.56E-04 |  | |
|  | NM_139033.2_bp1795_L | MAPK7 | 7.778336 | 6.72E-04 |  | |
|  | NM_139033.2_bp2933_L | MAPK7 | 1.50892 | 0.01269911 |  | |
|  |  |  |  |  |  | |
|  | NM_002752_bp482_L | MAPK9 | 1.218115 | 7.86E-16 |  | |
|  | NM_002752_bp629_L | MAPK9 | 1.083342 | 0.00946858 |  | |
|  | NM_002752_bp359_L | MAPK9 | 0.8979445 | 0.01727905 |  | |
|  |  |  |  |  |  | |
|  | NM_012224_bp2851_L | NEK1 | 0.7917204 | 0.006891274 |  | |
|  | NM_012224_bp1053_L | NEK1 | 0.7543465 | 0.006772207 |  | |
|  | NM_012224_bp4713_L | NEK1 | 1.057577 | 0.03122178 |  | |
|  |  |  |  |  |  | |
|  | NM_014397_bp524_L | NEK6 | 4.1749 | 0.001892672 |  | |
|  | NM_014397_bp769_L | NEK6 | 5.535319 | 0.03584691 |  | |
|  | NM_014397_bp967_L | NEK6 | 0.8770314 | 0.01473467 |  | |
|  |  |  |  |  |  | |
|  | NM_020529_bp915_L | NFKBIA | 0.6525248 | 1.69E-08 |  | |
|  | NM_020529_bp665_L | NFKBIA | 2.218079 | 0.003933312 |  | |
|  | NM_020529_bp338_L | NFKBIA | 1.529856 | 0.005628339 |  | |
|  |  |  |  |  |  | |
|  | NM_004688_bp682_L | NMI | 1.106179 | 0.03470464 |  | |
|  | NM_004688_bp608_L | NMI | 0.331133 | 0.03689144 |  | |
|  |  |  |  |  |  | |
|  | NM_006218_bp3025_L | PIK3CA | 0.8074465 | 0.005563584 |  | |
|  | NM_006218_bp1615_L | PIK3CA | 0.8086999 | 0.007004879 |  | |
|  |  |  |  |  |  | |
|  | NM_003156_bp975_L | STIM1 | 0.7321072 | 7.23E-05 |  | |
|  | NM_003156_bp2771_L | STIM1 | 0.6323433 | 1.15E-04 |  | |
|  | NM_003156_bp2574_L | STIM1 | 1.282521 | 0.01137614 |  | |
|  | NM_003156_bp929_L | STIM1 | 3.920376 | 0.04066691 |  | |
|  |  |  |  |  |  | |
|  | NM_003842_bp1321_L | TNFRSF10B | 2.321143 | 0.01430651 |  | |
|  |  |  |  |  |  | |
|  | NM_003318_bp2813_L | TTK | 1.137138 | 5.20E-09 |  | |
|  | NM_003318_bp2706_L | TTK | 0.8209573 | 2.71E-04 |  | |
|  | NM_003318_bp2746_L | TTK | 0.8476813 | 0.004585813 |  | |
|  |  |  |  |  |  | |
|  | NM_007284_bp224_L | TWF2 | 1.365139 | 0.002221342 |  | |
|  | NM_007284_bp1449_L | TWF2 | 1.130305 | 0.0013584 |  | |
|  | NM_007284_bp1060_L | TWF2 | 4.212272 | 0.03301523 |  | |
|  |  |  |  |  |  | |
|  | NM_020922.2_bp5236_L | WNK3 | 0.9824112 | 0.002552404 |  | |
|  | NM_020922.2_bp5621_L | WNK3 | 0.7488565 | 0.01301649 |  | |
|  | NM_020922.2_bp4793_L | WNK3 | 0.6986029 | 0.03493519 |  | |
|  |  |  |  |  |  | |
| **DAY 14 - shRNA with significant difference between IKKβ knockdown and control (p-value <0.05; Log fold >0.6)** | | | | | | |
|  | Refseq starting bp for shRNA | Gene symbol | Log_2_ fold | p-value |  | |
|  | NM_001080124_bp888_L | CASP8 | 1.122317 | 2.30225E-05 |  | |
|  | NM_001080124_bp2172_L | CASP8 | 1.273672 | 0.000317714 |  | |
|  | NM_001080124_bp1512_L | CASP8 | 0.7750598 | 5.6722E-09 |  | |
|  | NM_001080124_bp1583_L | CASP8 | 0.9626488 | 0.008189894 |  | |
|  | NM_001080124_bp787_L | CASP8 | 5.339176 | 0.04536617 |  | |
|  |  |  |  |  |  | |
|  | NM_001009565_bp843_L | CDKL4 | 1.926645 | 1.40E-21 |  | |
|  | NM_001009565_bp786_L | CDKL4 | 1.03047 | 3.96E-06 |  | |
|  | NM_001009565_bp535_L | CDKL4 | 0.8662986 | 6.39E-04 |  | |
|  |  |  |  |  |  | |
|  | NM_003582.2_bp1723_L | DYRK3 | 1.160252 | 8.79E-07 |  | |
|  | NM_003582.2_bp406_L | DYRK3 | 1.037968 | 8.71E-08 |  | |
|  | NM_003582.2_bp1297_L | DYRK3 | 0.894474 | 3.05E-07 |  | |
|  |  |  |  |  |  | |
|  | NM_001522_bp2514_L | GUCY2F | 2.469844 | 9.26E-04 |  | |
|  | NM_001522_bp3124_L | GUCY2F | 1.917024 | 0.003340621 |  | |
|  | NM_001522_bp2283_L | GUCY2F | 0.9325974 | 0.03637655 |  | |
|  |  |  |  |  |  | |
|  | NM_003299_bp924_L | HSP90B1 | 1.922238 | 0.001225676 |  | |
|  | NM_003299_bp2547_L | HSP90B1 | 0.6301904 | 1.53E-05 |  | |
|  | NM_003299_bp2115_L | HSP90B1 | 0.8436195 | 0.003299491 |  | |
|  |  |  |  |  |  | |
|  | NM_021798_bp2407_L | IL21R | 1.681169 | 1.24E-04 |  | |
|  | NM_021798_bp481_L | IL21R | 0.9342858 | 1.56E-20 |  | |
|  | NM_021798_bp972_L | IL21R | 0.6826626 | 0.04938783 |  | |
|  |  |  |  |  |  | |
|  | NM_004721_bp1233_L | MAP3K13 | 1.966979 | 1.24E-04 |  | |
|  | NM_004721_bp3074_L | MAP3K13 | 1.012289 | 1.27E-04 |  | |
|  | NM_004721_bp3189_L | MAP3K13 | 1.119352 | 0.0247307 |  | |
|  | NM_004721_bp3379_L | MAP3K13 | 2.002213 | 0.04911081 |  | |
|  |  |  |  |  |  | |
|  | NM_002969_bp733_L | MAPK12 | 0.8570277 | 1.24E-06 |  | |
|  | NM_002969_bp1109_L | MAPK12 | 0.8451509 | 2.11E-05 |  | |
|  | NM_002969_bp497_L | MAPK12 | 1.799857 | 0.01670559 |  | |
|  |  |  |  |  |  | |
|  | NM_017433_bp2857_L | MYO3A | 1.350347 | 1.97E-06 |  | |
|  | NM_017433_bp1867_L | MYO3A | 0.7275263 | 0.005292311 |  | |
|  | NM_017433_bp5199_L | MYO3A | 0.9401756 | 0.03362016 |  | |
|  |  |  |  |  |  | |
|  | NM_001001716_bp796_L | NFKBIB | 1.264067 | 6.34E-06 |  | |
|  | NM_001001716_bp1520_L | NFKBIB | 0.725054 | 0.01360574 |  | |
|  |  |  |  |  |  | |
|  | NM_006255_bp2646_L | PRKCH | 0.8816393 | 2.92E-05 |  | |
|  | NM_006255_bp389_L | PRKCH | 1.28983 | 0.003791171 |  | |
|  | NM_006255_bp1178_L | PRKCH | 0.9905972 | 0.01444106 |  | |
|  | NM_006255_bp2238_L | PRKCH | 0.7736247 | 0.0246028 |  | |
|  |  |  |  |  |  | |
|  | NM_170672_bp1360_L | RASGRP3 | 2.395582 | 0.004465111 |  | |
|  | NM_170672_bp2608_L | RASGRP3 | 0.6568853 | 1.35E-11 |  | |
|  | NM_170672_bp825_L | RASGRP3 | 1.18991 | 0.01381412 |  | |
|  | NM_170672_bp2411_L | RASGRP3 | 1.182128 | 0.02169342 |  | |
|  |  |  |  |  |  | |
|  | NM_006374_bp757_L | STK25 | 4.684028 | 1.30E-08 |  | |
|  | NM_006374_bp953_L | STK25 | 1.638297 | 9.29E-15 |  | |
|  | NM_006374_bp1985_L | STK25 | 0.9996464 | 0.0439085 |  | |
|  |  |  |  |  |  | |
|  | NM_006285_bp1416_L | TESK1 | 0.8702258 | 1.49E-08 |  | |
|  | NM_006285_bp1236_L | TESK1 | 1.979564 | 0.0114145 |  | |
|  | NM_006285_bp2317_L | TESK1 | 0.835487 | 8.47E-05 |  | |
|  |  |  |  |  |  | |
|  | NM_003842_bp3511_L | TNFRSF10B | 0.9014929 | 4.42E-08 |  | |
|  | NM_003842_bp822_L | TNFRSF10B | 1.534001 | 0.002019866 |  | |
|  | NM_003842_bp1557_L | TNFRSF10B | 1.281431 | 0.008540144 |  | |
|  | NM_003842_bp3120_L | TNFRSF10B | 0.6368372 | 0.001168683 |  | |
|  | NM_003842_bp1973_L | TNFRSF10B | 0.8378434 | 0.005951854 |  | |
|  |  |  |  |  |  | |
|  | NM_018979_bp7277_L | WNK1 | 2.039389 | 3.46E-12 |  | |
|  | NM_018979_bp6034_L | WNK1 | 0.9566902 | 2.53E-07 |  | |
|  | control_shRNA_WNK1 | WNK1 | 0.6827089 | 5.56E-17 |  | |
|  | NM_018979_bp6372_L | WNK1 | 1.085291 | 0.004345139 |  | |
