# Supplementary material for: A dual role for Caspase8 and NF-κB interactions in regulating apoptosis and necroptosis of ovarian cancer, with correlation to patient survival
Source: Cell Death Discov. 2015 Dec 14;1:15053–. doi: 10.1038/cddiscovery.2015.53 (PMC5198842; doi:10.1038/cddiscovery.2015.53)
Supplement: Supplementary Table S2 [file cddiscovery201553-s3.doc]

Supplementary Table 2: shRNA target sequences for CASPASE 8

| **Accession and target location** | **Oligo Name (forward target sequence)** | **RNAi Sequence** |
| --- | --- | --- |
| NM_001080124_bp888 | NM_001080124_bp888_F | GGGGATACTGTCTGATCATCA |
| NM_001080124_bp1512 | NM_001080124_bp1512_F | CCATCCTGACTGAAGTGAACT |
| NM_001080124_bp1583 | NM_001080124_bp1583_F | CAGCCTACTTTCACACTAAGA |
| NM_001080124_bp2172 | NM_001080124_bp2172_F | GGCTTATGATTCAGATTGTTA |
